# Memory Switches in Chemical Reaction Space

**DOI:** 10.1371/journal.pcbi.1000122

**Published:** 2008-07-18

**Authors:** Naren Ramakrishnan, Upinder S. Bhalla

**Affiliations:** 1 Department of Computer Science, Virginia Tech, Blacksburg, Virginia, United States of America; 2 Institute of Bioinformatics and Applied Biotechnology, Bangalore, India; 3 National Centre for Biological Sciences, Tata Institute of Fundamental Research, Bangalore, India; University of Washington, United States of America

## Abstract

Just as complex electronic circuits are built from simple Boolean gates, diverse biological functions, including signal transduction, differentiation, and stress response, frequently use biochemical switches as a functional module. A relatively small number of such switches have been described in the literature, and these exhibit considerable diversity in chemical topology. We asked if biochemical switches are indeed rare and if there are common chemical motifs and family relationships among such switches. We performed a systematic exploration of chemical reaction space by generating all possible stoichiometrically valid chemical configurations up to 3 molecules and 6 reactions and up to 4 molecules and 3 reactions. We used Monte Carlo sampling of parameter space for each such configuration to generate specific models and checked each model for switching properties. We found nearly 4,500 reaction topologies, or about 10% of our tested configurations, that demonstrate switching behavior. Commonly accepted topological features such as feedback were poor predictors of bistability, and we identified new reaction motifs that were likely to be found in switches. Furthermore, the discovered switches were related in that most of the larger configurations were derived from smaller ones by addition of one or more reactions. To explore even larger configurations, we developed two tools: the “bistabilizer,” which converts almost-bistable systems into bistable ones, and frequent motif mining, which helps rank untested configurations. Both of these tools increased the coverage of our library of bistable systems. Thus, our systematic exploration of chemical reaction space has produced a valuable resource for investigating the key signaling motif of bistability.

## Author Summary

How does a cell know what type of cell it is supposed to become? How do external chemical signals change the underlying “state” of the cell? How are response pathways triggered on the application of a stress? Such questions of differentiation, signal transduction, and stress response, while seemingly diverse, all pertain to the storage of state information, or “memory,” by biochemical switches. Just as a computer memory unit can store a bit of 0 or 1 through electrical signals, a biochemical switch can be in one of two states, where chemical signals are on or off. This lets the cell record the presence/absence of an environmental stimulus, the level of a signaling molecule, or the result of a cell fate decision. There are a small number of published ways by which a group of chemical reactions come together to realize a switch. We undertook an exhaustive computational exploration to see if chemical switches are indeed rare and found, surprisingly, that they are actually abundant, highly diverse, but related to one another. Our catalog of switches opens up new bioinformatics approaches to understanding cellular decision making and cellular memory.

## Introduction

Most chemical reaction systems have a single steady state, but a few interesting cases are known to oscillate [1], form spatial patterns [2], or have multiple stable states [3],[4]. Aside from their intrinsic mathematical and chemical significance, systems with multiple stable states are of particular biological interest because they can retain a “memory” of past inputs and cellular decisions [3],[4]. Bistability is a particularly interesting case of multi-stability, as it leads to switch-like behavior. Chemical stimuli can trigger a state change from one stable state to another. The current state of the chemical system is therefore a “memory” of this earlier stimulus.

A few biochemical switches have been extensively analyzed, including complex enzyme mechanisms [5],[6], kinase feedback [7],[8], dual phosphorylation [9], the cell cycle [10], triggering of caspases [11], and synaptic memory switches [12]–[14]. Two observations emerge from this set of known switches. First, relatively few switches are known. A recent computational exploration yielded only about 2% bistable models among those tested [15]. Furthermore, no entries are annotated as bistable in either KEGG (331 pathways) or BIOCARTA (355 pathways). Somewhat at odds with this absence of bistable pathways in pathway databases, kinetic models of bistable pathways are more common. There are several bistable models in the signaling model databases DOQCS (10/69; [16]) and BioModels.net (12/147; [17]), coming to about 10% of recorded models. This may be an overrepresentation, due to modeling interest in bistability. In particular, there are several signaling models that explore bistability as a basis for synaptic memory [12]–[14].

A second observation about the known bistable switches is that they are quite different in their chemical topologies. While feedback loops are a recurring motif [3],[18], there are some cases where enzyme saturation appears to play a role [13], and others where the balance between competing reactions itself generates bistability [9].

While signaling models tend to result in rather complex reaction systems, a distinct approach to the study of chemical bistability is driven from theoretical analyses of enzyme kinetics and flux reaction systems [3],[5]. These studies show that very few reactions are needed to achieve bistability. This raises the interesting question of whether there are core sets of reactions, or motifs, that are embedded in all bistable chemical reaction systems, despite their diversity. A corollary is whether such a set of motifs may help to detect bistable sub-systems in complex biological signaling networks.

Necessary conditions for bistability, such as positive loops in the system Jacobian, have been well characterized [18]. Earlier work by Clarke [19] parametrically defines all steady states of a given reaction system, but does not yield specific solutions when concentrations and rate constants are given. Recent studies detect chemical switches by testing for correlates of bistability [3],[5] or by looking for properties that frequently co-occur with bistability and, optionally, engineering bistability by minor modifications to such networks [15],[20],[21]. We sought to identify bistable systems without placing any “top-down” requirement on the mechanistic details, and use this unbiased search to reconstruct relationships between the switches.

Here we systematically explore chemical reaction space to show that bistable chemical switches are remarkably common. We show that all small bistable systems are related, and that larger ones frequently share motifs that may be predictive of bistability.

## Results

### Bistables Are Common

**Figure 1:**
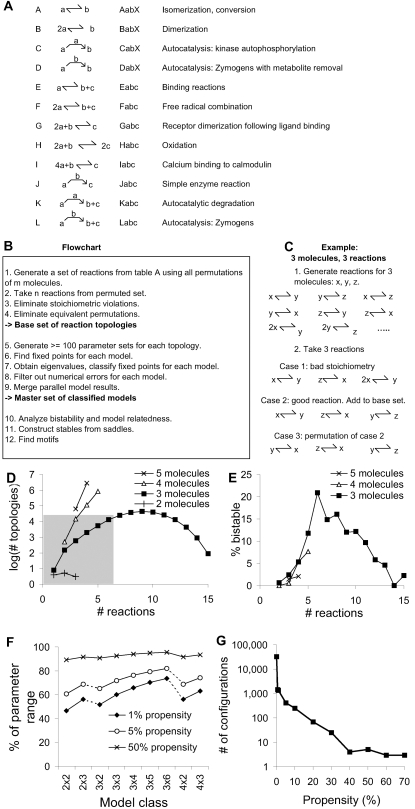
Finding Bistability. (A) Basis set of reactions with signatures and examples. Reactions C, D, J, K, and L are enzymatic and the enzyme name is at the bend of the arrow. (B) Flowchart for finding bistability. (C) Example of steps 1–4 in the flowchart. (D) Number of possible configurations rises steeply with increasing number of molecules and initially also with the number of reactions. Shaded region indicates configurations fully sampled in this study, the remainder were subsampled. (E) Bistable configurations initially become more common with increasing numbers of reactions, and for 3 molecules the percentage declines for more than 6 reactions. (F) Bistability must persist over a wide parameter range to be detected. Fraction of parameter range = ^#parameters^√(Propensity). Model class is expressed as m×n where m is the number of molecules and n is the number of reactions. (G) Frequency of occurrence of bistability as a function of propensity. Some configurations exhibit bistability over 30% of any parameter set in our selected range.

In our first phase of analysis, we began with a basis set of 12 reactions (Figure 1A) and systematically tested all reaction configurations involving 2 molecules, 3 molecules from 1 to 6 reactions, and 4 molecules from 1 to 3 reactions. In our second phase of analysis, we sampled a subset of possible reaction configurations involving 3 molecules from 7 to 15 reactions, 4 molecules from 4 to 5 reactions, and 5 molecules from 1 to 4 reactions. The number of possible configurations rose rapidly with the number of molecules and reactions (Figure 1D), and it took longer to test each configuration for bistability, hence we sampled a small subset of configurations for the second phase of analysis. For each configuration we generated ∼100 models using Monte Carlo assignment of concentration and rate parameters (see Methods section) and tested each for bistability. The propensity of a configuration for bistability was defined as the fraction of tested models for that configuration that exhibited two or more stable steady states.

We observed a large number of bistable systems even with our very sparse sampling of reaction parameter space (Figure 1E). 3,562 of the fully sampled configurations (∼10%) had at least one bistable model, and 918 of the larger reaction systems (∼5%) did. This large percentage was surprising for two reasons. First, known bistable configurations from biology are rare, as discussed above. Second, our sampling of parameter space was very sparse, so we would be likely to detect bistable configurations only if they remained bistable in a substantial portion of parameter space. Most known chemical bistable switches exhibit bistability in a relatively narrow range of parameters rarely exceeding a factor of two [9],[22]. While a factor of two may be substantial from a biological viewpoint, we required a 30-fold range to detect bistability. This was because even small models have a large number of parameters. For instance, a 3 molecule, 3 reaction system has 7 parameters. In order to obtain bistability in 1% of tested models of this configuration, bistability would have to be present over approximately half the sampling range (Figure 1F) for each parameter: (0.5)^7^∼0.01. Our logarithmic sampling spanned 3 orders of magnitude, so half this range is about 30-fold for each parameter. A few configurations had a propensity of over 50% (Figure 1G). This suggests that bistability in these systems is very robust.

Admittedly, due to our sparse sampling of parameter space, there could be undetected bistables in the space of systems sampled here. While a single configuration is sufficient to prove that a network has the capability for exhibiting bistability, our analysis methods do not support an impossibility proof for bistability. The range in which a system exhibits bistability can depend intricately on how the phase space is structured in terms of the system parameters such as molecule concentrations and rate constants. Bifurcation analysis can shed insight into parameter ranges feasible for realizing bistability. Nevertheless, even with the possibility of false negatives, it is significant that nearly 10% of explored systems are bistable and this percentage can only improve with greater analysis and exploration.

### Bistables Are Diverse

**Figure 2:**
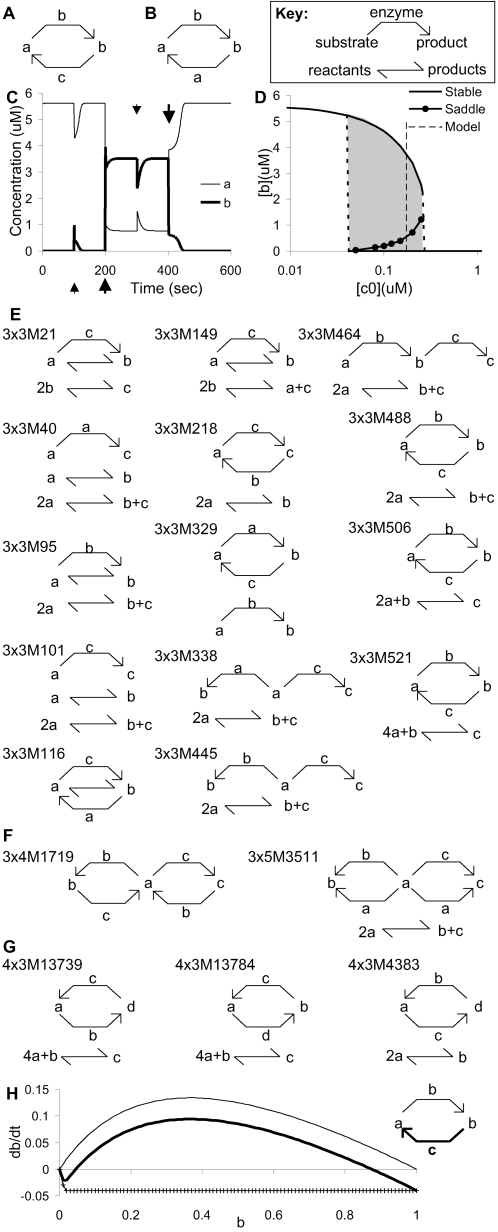
Example Bistable Models. (A) The simplest configuration, 3×2M101, i.e., having 3 molecules, 2 reactions, and being the 101st configuration in this class. (B) A simpler model that is bistable with Michaelis–Menten/Briggs–Haldane kinetics, but not with a mass action explicit representation of the enzyme-substrate complex. (C) Time-course of response of 3×2M101 to perturbations. (D) Stability diagram of 3×2M101, as a function of c0 (initial concentration of c). The bistable region is shaded. The specific model in panel A has c0 indicated by the dashed line. (E) All the 3×3 bistable configurations. (F) Two configurations with bistability propensity >70%. Model 3×3M445 in (E) is also over 70%. Note the model similarities and symmetry. (G) Three non-autocatalytic configurations with propensity 26%, 23%, and 16%, respectively. (H) Schematic of stability curve for an autocatalytic reaction (inset, thin arrow). There is a stable point at 0, and a saddle at 1. Addition of a rapidly saturating fast back-reaction (inset, thick arrow, and graph, crosses) converts this to a bistable model with the same configuration as (A). Now the stability curve (thick line) has a stable at 0, a saddle at ∼0.05, and another stable at ∼0.9.

The simplest bistable system (3×2M101) involved 3 molecules and 2 reactions (Figure 2A). We tested its switch-like behavior by introducing perturbations from its stable states (Figure 2C). Small perturbations in 3×2M101 (small arrows in Figure 2C) caused transients which return to the originating stable state whereas large perturbations (large arrows) caused state flips. An intuitively appealing simpler system with only 2 molecules (Figure 2B) turned out not to be bistable with our mass-action formulation for enzymes (Text S1).

**Figure 3:**
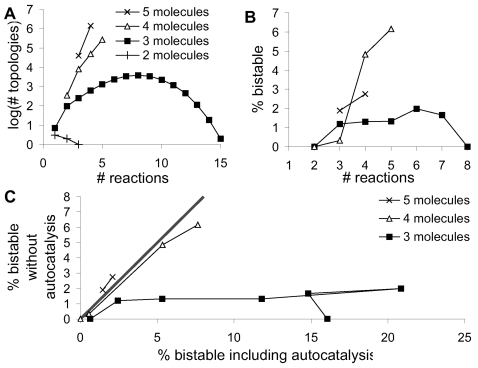
Effects of Autocatalysis. (A) Number of nonautocatalytic configurations as a function of number of reactions. (B) Percentage of bistable configurations for 3, 4, and 5 molecules as a function of number of reactions. Same symbols as in (A). (C) Comparison of bistability percentage for entire dataset (*x* axis) and set without autocatalysis (*y* axis). The thick line indicates equal percentages. 3-molecule systems have fewer bistables without autocatalysis, but 4 and 5 molecule systems have nearly equal bistables if autocatalytic reactions are excluded. The different points on each plot are for numbers of reactions. As bistability declines for large reaction numbers for 3 molecules, this curve folds back on itself.

Positive feedback loops, such as autocatalysis and catalytic loops, have been implicated as a common motif leading to bistability in signaling [3],[18],[23]. In our study, autocatalysis (reactions D and L) was frequently present in bistable models, but it was not necessary. When we excluded autocatalysis from small reaction systems with only 3 molecules, there were fewer possible reaction configurations and a ten-fold reduction in percentage of bistable configurations. However, autocatalysis had little effect on the percentage of bistable configurations for 4 and 5 molecules (Figure 3).

In addition to autocatalysis, we found several cases where bistability arose from more subtle chemical interactions (e.g., Figure 2G and 2F and 3×3M40 in Figure 2E). Such reaction sets would have been difficult to identify as bistable by searching for similarities to published networks [24],[25]. Interestingly, all our switches had exactly two stable states; the lack of higher levels of multi-stability may simply be due to our sparse sampling of parameter combinations.

### Uniqueness of Bistables

Are all discovered bistables distinct? Because isomorphisms were removed at the time of generating possible reaction signatures, we ensured that each discovered bistable mapped to a unique signature composed of the 12 basic reaction types. A remaining concern was that there might be equivalences in terms of the underlying dynamical system when the chemical systems were converted to mathematical models. We investigated this possibility by reducing all the composite reactions to approximate equivalences in the form of either a single reactant-single product reaction (type A) or a double reactant-single product reaction (type E) (see Methods section and Figure S2). We emphasize that these are “approximate” equivalences, for the following three reasons. First, many higher-order reactions required the inclusion of intermediate molecular species which were not present in the mathematical formulation of the original basis reactions. Second, the expanded reactions treated enzyme-substrate complexes as distinct molecular species having their own trajectories beginning from non-zero concentrations, whereas E-S complexes in the original reactions were initialized to zero in our modeling (see Methods). Third, backward rates from the E-S complexes to the reactants were assumed to be zero in our original modeling (e.g., for reactions C, D, J) whereas in the expanded modeling all reactions (forward and backward) have non-zero reaction rates. With these caveats in mind, we found situations where configurations were isomorphic according to our approximate mappings and both were bistable (Figure S2, frame B), and also cases where the configurations were approximately equivalent but one was bistable, and the others were not (Figure S2, frames C and D). These examples reveal that composite reactions such as are commonly used in biochemistry and in our study, complicate stability analysis in two ways. First, they may hide mechanistic similarities between systems. This can be addressed by expanding composite reactions into more basic steps, as we have done. Second, they may hide key assumptions such as intermediate species and fundamental reaction steps, which may cause major differences in the dynamical behavior of the reaction system. While this issue is important from a rigorous mathematical viewpoint, we point out that such approximations are inevitable when translating cellular biochemistry into idealized mathematical forms. We suggest that in many cases bistability is indeed preserved across approximations (e.g., Figure S2, frame B). Our study provides a framework for further systematic analysis of this question.

### Bistables Are Related

**Figure 4:**
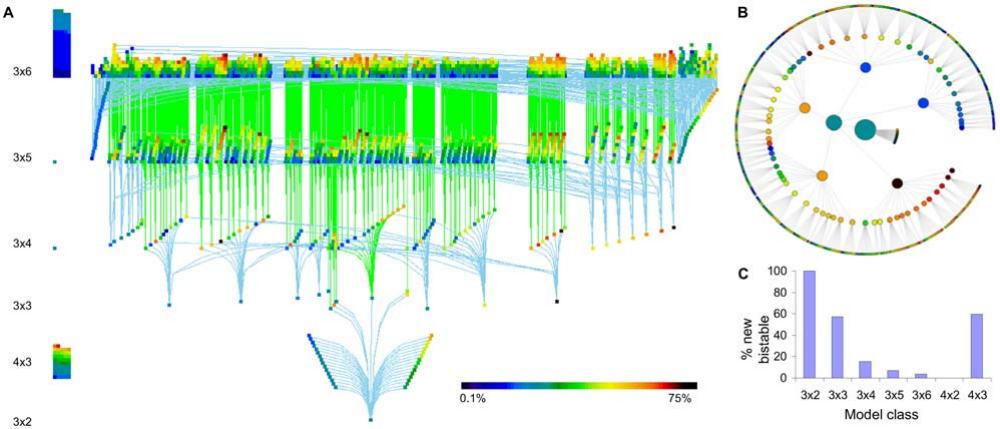
Bistability Runs in Families. (A,B) Representations of relationships between bistable configurations. Node color represents bistability propensity. Color scales for (A) and (B) are the same. (A) “Banyan tree” diagram showing multiple “root” bistable configurations that cannot be generated by addition of a single reaction to a smaller bistable configuration but are connected through larger configurations. Model classes are labeled on the left. Nodes are staggered vertically within bands for visualization. “Root” edges are in sky blue and deeper edges are in green. On the left are orphan models. (B) Minimum spanning tree rooted at 3×2M101. Inner nodes with smaller reaction sizes are drawn as larger circles. A few 3×3 bistables and the primarily low propensity systems they derive are not shown to minimize crowding. The “pie” denotes restriction of exploration of 4 molecule systems to only 3 reactions in this study. (C) The number of novel bistables drops sharply in larger reaction systems.

Does bistability “run” in families of related reaction topologies? To test this hypothesis, we constructed a directed acyclic graph (DAG) of configurations where each bistable configuration was a node, and each addition/removal of a reaction between nodes was an edge. We found that almost all bistable configurations from the first phase (3,415/3,562 = 95.9%) formed a single, highly interconnected set, i.e., a giant component. Most of the 147 “orphans” occurred at the boundaries of our sampling (98 at 3×6 and 47 at 4×3). These may simply represent novel ‘roots’ that connect further up in the reaction hierarchy. In Figure 4A, the DAG is represented as a multiply rooted “banyan-tree” like diagram where there is one main root (3×2M101) and multiple higher-order roots linked to the primary root through more complex, bistable “branches”. We may have missed low-propensity bistable configurations, so it is possible that isolated islands of lower propensity may be present. Conversely, it is also possible that finer sampling may uncover intermediate bistable systems that link orphan configurations into the DAG. We constructed a radial diagram restricted to those configurations that were derived from the simplest bistable configuration (3×2M101, Figure 4B). In Figure 4A and 4B, there was an apparent clustering of high-propensity nodes. We investigated this further by comparing projections of Figure 4A onto high-propensity nodes. A giant component persisted even when we increased the threshold for bistability propensity from >0 to ≥0.3 (Figure S1, frame A). This showed that highly bistable systems form a connected subgraph in the graph of all bistable systems. The much smaller non-autocatalytic subset of bistables was also multiply rooted with a giant component and a few separated nodes (Figure S1, frame B).

These graphs suggested that most bistable systems were derived from smaller ones. As reactions were added (Figure 4C), we encountered a decreasing number of novel bistables (i.e., cases that could not be derived by addition of a reaction to a smaller bistable configuration). This suggests that bistable systems involving small numbers of molecules may form the architectural core of more complex reaction networks that are also bistable.

### Relationship to Published Bistables

**Figure 5:**
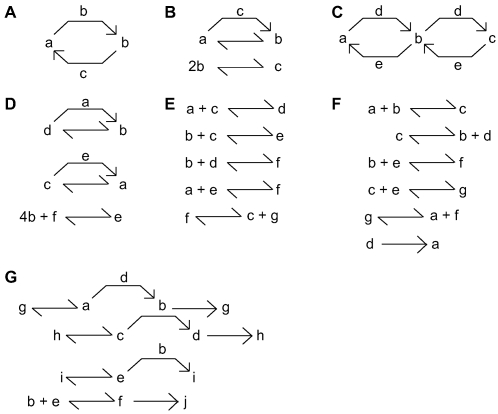
Known Bistables. (A,B) Published bistables from [20]. These are already present in our DAG. (C,D) Published bistables from [6] and [9]. These can be reduced to smaller models and have some bistable motifs from our dataset. (E,F) Bistables from [5]. (G) Bistable from [11]. None of (E), (F), and (G) had motifs from our dataset.

We tested two implications of the “bistables are related” observation. First, we asked if we could take a large published bistable system and remove one reaction at a time without losing bistability. If we could continue this process till we ended up at a bistable configuration present in our dataset, then we had a continuous trajectory from our known DAG of bistables to the published model. Second, we asked if the large bistable system was a superset of a known bistable configuration, without requiring that there were intermediate bistables between the two. We performed this analysis on several known bistable reaction systems from published work (Figure 5). We found that in four of these cases, the published bistables were either already among our catalog, or had a subset of reactions that was bistable. In the remaining three configurations, there was neither a connection between the published models to the tree, nor was there a subset of reactions that was bistable in the DAG.

We therefore hypothesized that the DAG of bistables may be nearly complete for small systems, but the increasing degrees of freedom afforded by greater numbers of molecules and reactions helped realize bistability in new, unseen, ways. We developed two analysis tools that work in complementary ways to explore such larger configurations.

### The “Bistabilizer”

A suggestive observation from our first phase was that a large fraction of configurations (∼60%) contained saddle points and line solutions (Text S1). Most of these saddles (80.8% of the non-bistable set) occurred when the concentration of all but one molecule in the system was zero. A simple example of this is in Figure 2H, where molecule **b** catalyzes its own formation from **a**. When **b** is at zero, **a** does not change – it is metastable. However, the addition of a small amount of **b** causes the system to ‘fall’ into a truly stable state where all molecules have been converted into **b**. As has been previously analyzed [20] a rapid but saturating back-reaction is one way to convert this into a true stable system. This can be done using an enzyme to remove **b** more rapidly than it builds up, at low levels of **b** (Figure 2H). In this case, we reconstruct our original simplest bistable system (compare Figure 2A and 2H). We developed an algorithm that introduced several reactions to achieve bistability using this approach, in a general but not necessarily minimal manner. Due to the added reaction complexity, we generated bistables from non-bistable systems involving only 3 molecules and up to 5 reactions. The bistabilizer added at least 2 molecules, 3 enzymes and a reaction for each converted saddle point. We were able to generate a 5-fold higher proportion of bistables than were present in the source configurations in a sample of 70,000 models. This construction may usefully complement bifurcation discovery tools [26] to generate and refine bistable configurations.

### Frequent Motif Mining

Our second tool used motif matching. We analyzed the configurations of smaller bistables plus the sparsely sampled larger bistables to find frequently occurring groups of reactions, and then searched for these motifs in unexplored configurations. We analyzed bistables in each configuration class (3 molecules, 4 molecules, 5 molecules) separately for frequent motifs. A motif must occur with sufficient frequency in the given class to be detected (see Text S1 for frequency thresholds used). Because motifs are subsets of chemical reaction systems, they may not quite have the same number of molecules as the class of systems from which they are mined; for instance, a motif mined from 5-molecule bistable systems may not itself have 5 molecules. Furthermore, observe that while a motif is a subset of reactions that is well-represented in bistable systems, it may not be bistable. We found the greatest number of motifs (1615) from 3-molecule systems, and smaller numbers for 4 and 5 molecules (143 and 28, respectively), probably because our initial harvesting of bistables in the larger systems had yielded fewer confirmed bistables to scan for motifs.

**Figure 6:**
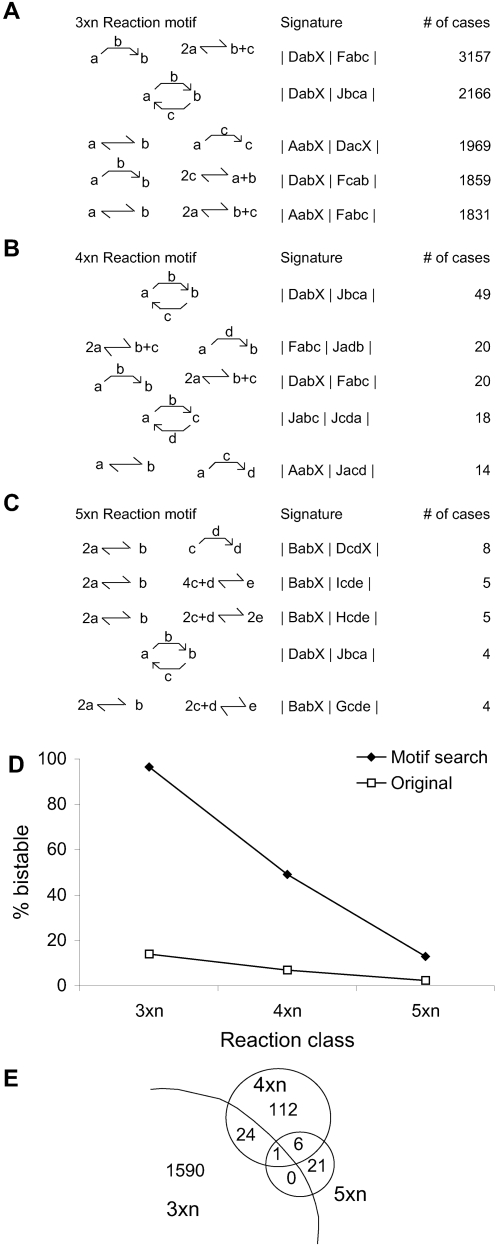
Motifs in Bistable Switches. (A–C) Top 5 motifs mined for 3-molecule, 4-molecule, and 5-molecule bistable systems. (D) Motifs improve search for bistables by about 7-fold, for each of the reaction classes tested. (E) Venn diagram of motifs among different classes of reaction configurations. The one motif shared by all classes of configurations is the same as the smallest bistable, model M101.

The motifs were mostly independent and only one motif occurred in all three reaction classes. Coincidentally, this common, two-reaction, motif (composed of reactions DabX and Jbca) was identical to a bistable found both in our analysis and in previous work ([20]; Figure 2A and 5A). This motif/bistable also occurred in the top-5 motifs mined for each class when the motifs were ranked by their frequencies (Figure 6A–C). Interestingly, four of the top-5 motifs in the 3 molecule case contained at least one of reaction D or reaction J and all of the top-5 motifs in the 4 molecule case contained at least one of these two reactions. In the case of 5 molecules, only two of the top-5 motifs involved either D or J, though our much smaller sample set may have led to skewed results in this case. The remaining top-5 motif among the 3 molecule systems and the three other top-5 motifs in the 5-molecule systems utilized basic reversible reaction types such as A, B, F, G, H, and I that did not involve autocatalysis or even basic enzyme catalysis.

Just as a motif occurred in multiple bistable systems, a given bistable system could exhibit many distinct motifs. We used this property to advantage to help rank untested configurations for their potential to exhibit bistability. In each configuration class (3-molecule, 4-molecule, or 5-molecule systems), we searched for motifs specific to that class, and ranked the (untested) configurations in terms of the number of motifs they exhibited. We evaluated ≥100 of the top configurations for each class, exploring 120 parameter sets for each. We found bistability in 96% of the 3 molecule systems (214/222), 49% of the 4 molecule systems (49/100), and 13% of the 5 molecule systems (82/641). These numbers significantly improved upon the random sampling results of the second phase of analysis (Figure 6D).

Finally, we compared the motifs across the three classes (3, 4, and 5 molecule systems) to investigate whether there were any overlaps in mechanisms by which 3-molecule, 4-molecule, and 5-molecule systems exhibit bistability. On face value, there was little overlap between the motif sets taken pairwise (Figure 6E). To understand the distinctions better, we searched for all three sets of motifs in all three classes of bistable systems. For each motif, we counted its frequency in each class and identified the absolute value of the difference in frequencies across classes. The median difference in frequency is cataloged in Table 1 which shows that the motifs in 4-molecule systems were more similar to those in 5-molecule systems than either to 3-molecule systems. This suggests that there are qualitatively different mechanisms for bistability vis-à-vis 3 molecule systems and higher molecular systems.

**Table 1:** Median Difference in Frequency of Motif Sets Searched across Different Classes of Bistable Systems.

	3xn	4xn	5xn
3xn	–	15%	29%
4xn	21.5%	–	1.6%
5xn	45%	3.5%	–

Rows: Motif set. Columns: Class of bistable systems.

## Discussion

Our study draws the first stability map of chemical reaction space. We find that bistables are common, especially in smaller reaction systems. They are also very robust, i.e., we find many configurations that are bistable over a very wide parameter range. Smaller bistables are all related to each other in a tree-like manner. While the overall configurations that support bistability are very diverse, there are frequently recurring motifs of reaction groupings in such configurations. These motifs serve to identify promising candidates in higher order systems.

### A Resource for Studying Bistability

Signaling motifs have been regarded as a good way to abstract out the chemical complexity of signaling [4],[24]. Specifically, positive feedback loops have long been considered good indicators for bistability. Our study shows that such broad network features are inadequate. The simplest form of positive feedback, that is, autocatalysis, is a good predictor for bistability only in very small reaction sets. In reactions with 4 or 5 molecules the proportion of bistables does not seem to depend on the presence or absence of autocatalysis (Figure 3). Instead we propose that our library of bistable configurations is a more complete and stronger approach. Our catalog, available from the DOCSS (Database of Chemical Stability Space) website at http://docss.ncbs.res.in, provides complete model descriptions in chemical reaction signature format, as well as selected bistables in SBML format. Together with recent methods to reduce chemical networks to their core reactions [27],[28], our catalog may open up chemical and bioinformatics approaches to searching for bistability in biochemical signaling pathways.

### Evolutionary Implications

Bistable switches are important in biology in maintaining cellular history and decisions. Our study shows that there is a large repertoire of such switches for natural selection to draw upon, including many very simple switches. Furthermore, several of these switches are highly robust with respect to parameter variations. This has two implications for evolution. First, it is easy for evolving biochemical networks to stumble upon parameters that will give a switch. Second, such switches themselves will work effectively over a wide range of parameter conditions. The relatedness of the switches through addition or removal of individual reactions is also a good substrate for evolutionary modification. For example, a mutation that adds another enzyme regulator to a bistable switch is, by this argument, quite likely to retain the original bistability, along with the new regulatory properties. Overall, our survey of chemical topologies hints at an interconnected and rather well-populated terrain of bistability in a biologically biased region of chemical space.

## Methods

### Modeling Chemical Reaction Space

We selected a set of 12 primary chemical reaction steps (Figure 1A) as the “alphabet” from which we performed our exhaustive search of chemical reaction space. In principle a small set of reactions may suffice to build up to arbitrary reaction schemes. For instance, using two reactions of type E
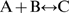
we can realize the higher order enzymatic reaction
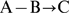
which is reaction J in our system, by modeling it as a composite of
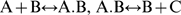



Our choice of primary chemical steps was biologically-inspired. In other words, we found a different proportion of bistables than we would see if we used, say, only the most elementary reactions such as type A and type E (Figure S2). Instead we reduced the parameter space by using biologically-inspired composite reactions, and hence sampled more completely in biological chemistry space. From this set of 12 primary chemical reaction steps, we constructed all possible reaction configurations involving 2 or 3 molecules, 4 molecules up to 5 reactions, and 5 molecules up to 4 reactions. This was a total of ∼2,800,000 configurations. These reaction configurations were topological: they defined the molecules and chemical steps, but did not specify concentrations or kinetic parameters.

We constructed reaction architectures involving *m* molecules as follows. We first selected one reaction out of the set of 12 reactions. We then assigned molecules to the slots of the reaction. For example, the reaction *J* has three slots, so we could assign molecules a, b, and c to this reaction. Having set up the first reaction, we then repeated the process *n* – 1 times to obtain a configuration of *m* molecules and *n* reactions (Figure 1). We eliminated all stoichiometrically invalid configurations by row-reducing the augmented stoichiometric matrix and checking for conserved moieties [29]. We repeated this entire process for all possible permutations of the *m* molecules. Similar approaches have been employed at a more elementary level of chemical reactive species to computationally analyze reaction systems [30],[31].

### Signature Typing

We signed each reaction with a terse unique 4-character string that completely specified all reactants and products, so that the first character of a reaction signature denotes one of the 12 reaction types (A–L), and the remaining two or three characters denote the molecular species participating in various roles in the reaction. The signature for a reaction architecture was obtained by concatenating the signatures for the constitutent reactions. We checked for isomorphic signatures (see Text S1) and only one signature per unique system was retained. The number of such unique, stoichiometrically valid reaction architectures was combinatorially large (Figure 1D). As further reactions were added, the number of possible configurations peaked and then declined because of stoichiometric constraints and symmetry (Figure 1D).

Our set of configurations did not deal with two cases that have previously been analyzed for bistability: continuous flux and buffered systems [21]. Instead our reactions required that there was mass conservation among the named molecules, but did permit the presence of ‘hidden’ molecules that were folded into the rate terms. Thus we represented a kinase reaction as an elementary enzymatic step, by “hiding” the ATP and ADP exchange: Substrate–Kinase → Product.

In this manner our reaction systems also accommodated steady state cases where continuous metabolic input was necessary to sustain stability. We stipulated that these ‘hidden’ molecules were stoichiometrically balanced within individual reactions, such as the enzymatic step above. We were able to approximate many cases of buffering simply by having a high concentration of the ‘buffered’ species.

### Parameterization

In order to assess bistability we needed to work with specific models, with all parameters specified. We generated at least 100 models for each configuration we tested. Each model was generated from one of the configurations using Monte Carlo sampling to assign rate constants and concentrations. We chose concentrations using logarithmic sampling in the range 10 nM to 10 µM. This spans the concentration range of most biochemical reagents. We chose rate constants using logarithmic sampling in the range 0.01 µM^−*N*^ s^−1^ to 10 µM^−*N*^ s^−1^, where *N* was the order of the reaction from 0 to 4. Again, these rates were chosen to span the common range of biochemical reaction parameters.

Due to computational limitations we sampled only the smaller reaction sets completely for bistability. We completely sampled all configurations with 2 molecules, 3 molecules up to 6 reactions, and 4 molecules up to 3 reactions (Figure 1A–C and Text S1). This amounted to a total of 100,000 configurations and ∼20e6 models, sampling at least 100 models per configuration. We sampled the remaining reaction sets more sparsely, mostly 1 in 100, but we used 1 in 1,000 sampling for the very large 4×5 and 5×4 reaction sets.

### Identifying Steady States

We found steady states for each model using two distinct methods: homotopy continuation [32] and time course analysis (Text S1). Briefly, homotopy continuation finds steady states by tracking solution paths of systems of simultaneous equations. Time-course analysis simulates models from a number of distinct initial conditions toward steady states. Neither of these methods determined the global bifurcation behavior of the reaction system: they only identified fixed points of the fully parameterized model. We classified solutions as *stable*, *saddle*, or *other* using eigenvalue calculation and simulation around steady states (Text S1).

## Supporting Information

Text S1 Supplementary Information about Methods and Algorithms To Accompany the Main Text of the Paper.
(0.11 MB DOC)

Figure S1 Minimum Spanning Tree (A) and Banyan Tree Graph (B). (A) Minimum spanning tree derived from high propensity configurations (rooted at 3×3M445). Inner nodes with smaller reaction sizes are drawn as larger circles. Four levels of the giant component are shown that shrinks but remains connected as the propensity threshold is raised. Dark blue is the range 0<propensity<0.1, white is propensity ≥0.3. (B) “Banyan tree” graph of nonautocatalytic models, with a few orphan systems. Color scale indicates propensity of configurations.
(1.29 MB EPS)

Figure S2 Isomorphic Mappings. (A) Table of Isomorphic Mappings from the Original 12-Reaction Set to a Minimal Set Consisting of Reactions A and E. Note that many mappings require the formation of intermediate molecular species and that enzyme reactions become bidirectional during the expansion. (B) An example of an isomorphic mapping where bistability is preserved, despite the change from unidirectional enzyme to two bidirectional conversion reactions. (C,D) Examples of isomorphic mappings that lose bistability. In both cases, the bistability is lost because the expanded form of an enzyme contains two bidirectional reactions, which can also be mapped to an enzyme with the reverse direction.
(0.24 MB EPS)
