# Supplementary material for: Memory Switches in Chemical Reaction Space
Source: PLoS Comput Biol. 2008 Jul 18;4(7):e1000122. doi: 10.1371/journal.pcbi.1000122 (PMC2440819; doi:10.1371/journal.pcbi.1000122)
Supplement: Text S1 — Supplementary Information about Methods and Algorithms To Accompany the Main Text of the Paper. (0.11 MB DOC) [file pcbi.1000122.s001.doc]

## Memory Switches in Chemical Reaction Space

Naren Ramakrishnan^1^ and Upinder S. Bhalla^2^

## Supplementary Information

**Definitions:**

Reaction set: A group of stoichiometrically consistent chemical reactions with m molecules and n reactions.

Topology: The underlying (directed) graph structure of a set of molecules involved in a reaction set.

Reaction architecture, configuration: Instance of a reaction set involving specific molecules. For a given reaction set, there is a defined topology, but many reaction configurations depending on the specific molecules used.

mxn: A configuration class denoting the set of possible systems having “m” molecules and “n” reactions.

Motif: Subset of a reaction architecture/configuration that recurs frequently in systems of interest.

Model: An instance of a reaction architecture with specific chemical rates.

Signature: A character string that uniquely identifies specific reaction architecture. Optionally can be extended with parameters to fully specify a model.

Steady state: A fixed point of the dynamical system of a chemical model, i.e., a set of values for chemical concentrations at which the time derivatives of all species are zero. Also called a critical point.

Linear stability analysis: The approach of analysing stability properties of steady states in a higher order system by linear zing the system around the steady state of interest and characterizing the eigenvalues of the linearization.

Stable point: A steady state that is asymptotically stable, i.e., where all perturbations within a neighbourhood decay. True when real parts of all eigenvalues in the linearization are negative.

Unstable point: A steady state where all perturbations result in unbounded amplifications of molecular concentrations. True when real parts of all eigenvalues in the linearization are positive.

Saddle point: A steady state where real parts of some eigenvalues in the linearization are positive and real parts of some others are negative. True when there are basins of attraction as well as repulsion around the steady state. Also described as a steady state for which there are trajectories that tend to the steady state in both positive and negative time, or a metastable state.

Saddle point at zero: A saddle point where concentrations of molecular species are zero.

Multi-stability: Existence of two or more (asymptotically) stable steady states.

Stabilizer reactions: A set of reactions that can convert a saddle point at zero concentration into a stable point.

Propensity for multi-stability: % of multi-stable models of the same reaction architecture.

## Methods

### Generation of architectures

We used a set of 12 primary reactions (Figure 1). In principle a smaller subset may be sufficient to obtain all possible architectures (e.g., reactions A and E of Figure 1A) but we included additional composite reactions such as enzyme-catalyzed and implicit ATP-driven irreversible steps so as to search more deeply into biologically relevant reaction schemes. For instance, each enzymatic reaction is mathematically represented as

E + S <==> E.S ---> E + P

which can be derived from a reaction of type E, and a reaction of type A, if the backward rate for this reaction is set to zero. An additional molecular species to represent the enzyme-substrate (E-S) complex is also needed if we are to represent the enzyme in terms of elementary reactions. Supplementary Fig. 2 depicts mappings for all our basic reactions in terms of types A and E. These equivalences are approximate since they introduce additional molecular species and require at least two other simplifying assumptions (see main text).

We constructed reaction architectures involving *m* molecules by systematically pulling a reaction out of the set of 12 reactions, permuting through all possible combinations of the *m* molecules, and repeating *n* times to obtain a configuration of *m* molecules and *n* reactions (Figure 1 in the main text). We eliminated all stoichiometrically invalid configurations by row-reducing the augmented stoichiometric matrix and checking for conserved moieties [1]. Similar approaches have been employed at a more elementary level of chemical reactive species to computationally analyse reaction systems[2,3].

In the first phase of our study, we exhaustively surveyed all possible reaction architectures involving 2 molecules, 3 molecules up to 6 reactions, and 4 molecules up to 3 reactions (Figure 1 flowchart).

For the purposes of our classification, *m* molecules refers only to number of initial molecules, not to the E-S complexes. However, in our calculations we explicitly compute values for the E-S complexes as well. All E-S complexes were initialised to a concentration of zero. Our reactants could be proteins, isomers, ions, polymers or any other distinguishable molecular species, so our analysis is fairly general.

### Signature Typing

We signed each reaction with a terse unique 4-character string that completely specified all reactants and products, so that the first character of a reaction signature denotes one of the 12 reaction types (A-L), and the remaining three characters denote the molecular species participating in various roles in the reaction. For reactions with only two species, the fourth character is denoted as ‘X’. For instance, the reaction **2a <=> b** is typed as “BabX” whereas the reaction **2b <=> a** is typed as “BbaX”. Similarly, the enzymatic reaction **a ---b---> b+c** (i.e. **a** is catalyzed by **b** to form **b + c**) is typed as “Labc”. This gives rise to a basis set of 7 reactions involving two molecules and 47 reactions involving three molecules. Signatures for a reaction architecture are obtained by concatenating the 4-character strings corresponding to the constituent reactions.

### Isomorphism Checks and Pruning

The set of all signatures for a given number of molecules and reactions is then processed as a whole to identify disconnected and redundant networks which can be pruned. An example of a disconnected network is the reaction set comprised of reactions A<=>B and C<=>D). The problem of redundancy arises due to isomorphic mappings between architectures. We identify these mappings and use them to retain only one architecture per topology. For instance, the architectures “AabX” and “AbaX” have the same topology (i.e., **a** **<=> b**) and, in such cases, the architecture with the lexicographically smallest string is utilized (here, “AabX”). Similarly, “AabXCbaX” is eliminated in favor of “AabXCabX”.

There are three forms of isomorphisms at work in reaction signatures. First, the order in which reactions are listed gives rise to a simple form of isomorphism. For instance, “AbaXAcaX” is the same as “AcaXAbaX”. Second, some reaction types (specifically, A, E, F, K from Figure 1) can each be stated in two ways. For instance, AabX is the same as AbaX. Similarly, Kabc is the same as Kacb (but not Kbca). But other reaction types do not offer such rotations. For instance, BabX cannot be substituted for BbaX in a configuration (modulo a caveat coming below). Finally, the molecule names (like “a”, “b”) are simply placeholders, so shuffling the names around does not give rise to a new system, as long as the changes are universally made. Thus, the single-reaction system BabX is basically the same as BbaX, but not if other reactions are present. To trap isomorphisms, we map every generated signature to a unique, canonical, lexicographically smallest signature. Given a signature, we apply all of the above rotational transformations (i.e., permute the order of reactions, apply equivalences for A, E, F, and K, and rotate molecule ids) and generate a master list of equivalent signatures. We sort the signatures from the master list and pick the smallest signature as the canonical form. Two signatures are then isomorphic if they map to the same lexicographically smallest signature.

The cost to implementing these isomorphisms can be calculated as follows. For a configuration with “m” species, “n” reactions (of which “p” reactions fall into categories A, E, F, or K), the number of equivalent signatures is: (n!*m!*2^p). Since the number of species considered in this study is small, the major sources of isomorphisms are reactions. We implement the isomorphism checks as string operations rather than in the general setting of chemical reaction networks as graphs, although essentially the underlying problem is one of (directed) graph isomorphism. This problem is computationally complex and although efficient algorithms exist, it is not proven to be either in P (polynomial time solvable) or in the NP-complete class of intractable problems. Table 1 depicts, for each configuration class, the amount of time required to implement the isomorphism checks and its relation to the time required to generate the signatures. These times were recorded on a desktop workstation with an Intel Core 2 Duo 1.8GHz processor (1GB RAM). The larger configuration classes were generated and pruned on a parallel cluster of workstations. Table 1 reveals that the bulk of the time is devoted to removing isomorphism checks and that it very quickly overshadows the time to generate signatures.

Table 1: Time taken to generate signatures and implement isomorphism checks (seconds).

| Configuration class | Number of generated signatures | Time taken to generate signatures (seconds) | Number of signatures after isomorphism checks | Time taken to implement isomorphism checks (seconds) |
| --- | --- | --- | --- | --- |
| 2xn | 15 | 0.0008 | 12 | 0.001796 |
| 3x2 | 205 | 0.02 | 156 | 0.052 |
| 3x3 | 891 | 0.35 | 576 | 0.523 |
| 3x4 | 3690 | 2.28 | 1920 | 4.882 |
| 3x5 | 12854 | 11.78 | 5457 | 40.437 |
| 3x6 | 36975 | 49.43 | 13052 | 260.828 |
| 3x7 | 83141 | 170.5 | 25059 | 1293.5 |
| 3x8 | 144166 | 441.64 | 37764 | 6847.41 |
| 4x2 | 671 | 0.3 | 496 | 1.07 |
| 4x3 | 22751 | 16.54 | 14298 | 121.61 |

Even after applying isomorphism checks, the number of such unique, stoichiometrically valid reaction architectures is combinatorially large (Table 2). As further reactions are added, however, the number of possible configurations peaks and then declines because of stoichiometric constraints and symmetry (Figure 1 in the main text).

Table 2: Number of unique configurations for each configuration class studied in this paper.

| **Configuration class** | **Number of unique configurations** |
| --- | --- |
| 2xn | 12 |
| 3x1 | 8 |
| 3x2 | 148 |
| 3x3 | 576 |
| 3x4 | 1920 |
| 3x5 | 5457 |
| 3x6 | 13052 |
| 3x7 | 25059 |
| 3x8 | 37764 |
| 3x9 | 44162 |
| 3x10 | 39524 |
| 3x11 | 26592 |
| 3x12 | 13020 |
| 3x13 | 4368 |
| 3x14 | 904 |
| 3x15 | 88 |
| 4x2 | 496 |
| 4x3 | 14298 |
| 4x4 | 114751 |
| 4x5 | 825513 |
| 5x3 | 65245 |
| 5x4 | 2813265 |

### Parameterization

For each reaction architecture, we defined *m + 2n* rate parameters: one initial concentration term for each molecular species, and two rate terms for each of the reactions. Each binding/conversion reaction has a *Kf* and a *Kb* term, and each enzymatic reaction has a *kcat* and *Km* term. We used logarithmically distributed random-number sampling to pick concentration terms between 0.01 and 10 uM, and rate terms between 0.01 and 10 /sec. Higher order reactions were sampled between the same ranges with correspondingly higher concentration units, e.g., 0.01 to 10 /sec/uM. These values were chosen to fit in the range of most biological reaction parameters. We assumed all reaction loops had hidden terms for thermodynamic balance. For example, all enzyme reactions have an irreversible final step which is justified on the grounds of hidden molecules with a high fixed gradient, such as ATP:ADP for kinases. We extended this assumption to the occasional reaction loops involving multiple reversible reactions, though in principle we could have used detailed balance to reduce the parameters in such systems. In this manner we parameterized >= 100 specific instances of each architecture for further analysis.

### Finding fixed points

We then asked: what are the fixed points of each model? Such fixed points define the stable, unstable, and saddle states of each model in terms of concentrations of each species. We determined fixed points in two ways.

First, we implemented a rigorous root finder (Homotopy Steady State Finder, HSSF) based on homotopy continuation methods for polynomial systems [4]. Given the dynamical system x’ = F(x), this method finds steady states by fixing the rate of change of each molecule species to zero and solving for x in F(x) = 0. The simple approach of writing out differential equations for each molecule is not sufficient because chemical systems obey mass conservation, so the set of equations is not independent. However, the same chemical conservation laws also provide additional constraints based on the initial concentrations of molecules. We used conventional stoichiometry matrix calculations [1] to generate the minimal set of differential equations and conservation laws. This results in a set of polynomial, nonlinear equations suitable for solution by homotopy methods. These were slow but reliable and accurate in most cases.

Homotopy methods first define a homotopy map (1-α) G(x) + αF(x) that interpolates between F(x) and a suitably designed, easier to solve, ‘start system’, G(x). The continuation process then involves tracking the solutions (zeros) of G(x) (when αis 0) in complex projective space to find the zeros of F(x) (when αis 1). We primarily utilized codes that find isolated solutions although software to determine manifolds also exist (see discussion later about “line solutions”). All isolated mathematical solutions determined by such homotopy methods are then screened for chemical validity, i.e., to ensure that they have purely real solution components and non-negative molecule concentrations. We also pay attention to recording the multiplicities of the solutions (cycle numbers, determined by number of homotopy curve paths leading into a given solution) and their zeroness (number of solution components equal to zero).

For a small fraction of systems, the homotopy codes were unable to find solutions, primarily because the homotopy curve was not trackable with sufficient stringency (set at 10^-8^) , or the built-in iterative root finders failed to converge, even after 10^4^ iterations. Therefore, we developed a second approach-time-course analysis of steady states (TCAS)-that simulated models using different initial concentrations satisfying conservation laws, and used heuristics to identify steady states. The heuristics were to run the simulation out for a fixed time (600 sec) and use exponential extrapolation to estimate the solution value at infinity. This was fast but prone to missing saddle points as well as generation of spurious solutions in some circumstances. However, it succeeded in identifying numerous line solutions and saddle cases that caused difficulties for HSSF and therefore served as a valuable cross-check. The HSSF and TCAS calculations, for ~8.3 million models, took a total of approximately 100 CPU years on three clusters at VT and NCBS.

### Classification of solutions

We classified the solutions as stable, unstable, and saddle points by first eliminating dependent species in the chemical system, thus reducing the dimension of the system, linearizing the reduced system around the steady state to obtain the Jacobian of the chemical system, and then assessing the signs of the real parts of the eigenvalues of the Jacobian. A steady state with all negative real components is an asymptotically stable state (the imaginary components dictate whether the system will exhibit spiral or line trajectories toward the steady state) whereas one with both positive and negative real components is a saddle state. The case of all positive real components was vanishingly rare (only 23 from the millions of models tested, all involving 4 molecules) and denotes a steady state that is unstable under all perturbations. In assessing negativity of component values, in addition to examining signs, we required that the absolute value of the eigenvalue be greater than 10^-4^. Conclusions derived from the linearized system in this manner carry over to the original system as long as we focus on establishing only stable, unstable, or saddle properties (in contrast to degenerate properties such as centres and non-isolated fixed points, which result when at least one eigenvalue has real part zero). While this method is mathematically rigorous, numerical errors and non-normal matrices led to failure of this analysis in about 1% of cases. As a cross-check, we also implemented a heuristic method to determine if solutions were stable based on the simulated time-evolution of the model following perturbation. In this method we ran simulations for 1000 sec of simulated time, with starting concentrations of all molecules taken from the solutions. We first checked if the solution itself remained steady. We then introduced perturbations into each initial molecule concentration in turn, and repeated the process. In each case we estimated the normalized dot product (cosine of the angle between the starting concentration vector, and the final vector). If this was >= 0.999, we regarded the solution as stable. Molecule perturbations were 1% of the initial value if over 1.0, and 1% of the total initial concentration of molecules if under 1.0.

When using the steady-state method, we frequently encountered multiple plausible solutions. Most of these were identified as line solutions. This was done by taking pairs of solutions, and asking if any of the other solutions were linear combinations of them. If so, all the linear solutions were classified as a line solution. Rigorous homotopy codes to find solution manifolds can be utilized to characterize such solutions algebraically.

As a further heuristic, we stipulated that all solutions classified as bistable should have two stable points and at least one saddle point.

Together, our criteria are fairly stringent and we are likely to have eliminated a large number of valid bistable solutions. Our goal was to minimize the number of falsely classified bistables. This stage results in 3483 3-molecule and 79 4-molecule bistable configurations.

**Graphing the space of chemical reaction networks**

We view the space of reaction architectures as a directed graph **G** where an edge from node **a** to node **b** indicates a stoichiometric extension of architecture **a** into **b,** by addition of a single reaction and possibly an additional molecule. Note that **G** can be interpreted as a partial order with the twelve (incomparable) primary reactions occupying the lower frontier. The nodes of **G** are coloured according to their propensity and we visualize **G** in three ways: by projecting it onto all bistable architectures (Fig. 4A in main text), those bistable architectures derivable from the simplest model (3X2M101; see Fig. 4B in main text), architectures with high propensity for bistability (supplementary Fig. 1A) and those that involve only non-autocatalytic reactions (supplementary Fig. 1B).

# Estimating saturation of enzymes

Saturation S was calculated as

S = [complex]/[enz] = ( [enz] * [sub] / (Km + [sub]) ) / [enz]

= [sub] / (Km + [sub])

where [complex] is concentration of enzyme-substrate complex, [sub] is substrate concentration, and [enz] is enzyme concentration, and Km is the Michaelis-Menten constant for the enzyme.

S is between 0 and 1, and is 1 for a completely saturated enzyme.

This calculation was applied to each of the stable solution (steady) states of each model that had at least one enzyme. If the model had multiple enzymes or multiple stable states, the highest value of saturation was used.

### Parameter statistics

We wished to check if our choice of kinetic parameter ranges introduced a bias or restricted our sampling of bistables. We looked into this by measuring the geometric mean and standard deviation of all kinetic parameters of bistables. Since the sampling was logarithmic, the geometric mean was an appropriate measure. We found that in most reactions the geometric mean was near the middle of the sample range of 0.01 to 10: √( 0.01 * 10 ) ~ 0.31, and the geometric standard deviation was around 7. This suggested that for these parameters, bistability occurred for a large fraction of the sampled range, without bias. For a few parameters the geometric mean differed from this value, suggesting that here our kinetic parameter range may have restricted our ability to find bistables. For example, the kb for reaction F was consistently near 0.6 uM^-1^s^-1^ for all the 3-molecule bistables. Another outlier was Km for reaction D, which was 0.7 uM for the 4x3 bistables. Nevertheless, even in these cases the mean was around a factor of two away from the middle of the sample range. We interpret this to mean that our parameter range was broad enough to sample adequately for all parameters.

# Second Phase of Analysis

For the second phase of analysis, to explore larger systems, we sampled both configurations and model parameters in each configuration (100 each as before). Specifically, we sampled a subset of possible reaction configurations involving 3 molecules from 7 reactions and up to 15 reactions, 4 molecules from 4 to 5 reactions, and 5 molecules up to 4 reactions. Every kth configuration was selected for testing for bistability, where “k” varies depending upon the configuration class. We first sorted the configurations by their reaction signatures (after rendering them in lexicographically smallest, canonical, form) and picked every kth system from the sorted list. Thus, although not every system had a non-zero probability of being picked, the sample broadly reflects the distribution of reaction types as observed in the complete set. For instance, about 10% of the systems in the (5 molecule, 4 reaction) class involve at least one “A” type reaction and the remaining 90% do not involve any “A” type reaction at all. These percentages are reflected in the sampled subset of configurations as well. Similarly, statistics for combinations of reaction types that form the prefixes of reaction systems are broadly maintained.

Repeating the analysis steps as before gives 753 new 3-molecule bistable systems, 87 new 4-molecule bistables, and 71 5-molecule bistables.

# The “Bistabilizer”: Constructing a Bistabilizing Set of Reactions

The bistability construction algorithm changes reaction networks with the goal of converting saddle points into stable points. It works especially on saddle points with high zeroness, that is, saddle points where many of the molecules are at concentration 0.

Analysis:

If we have a reaction such as

A

X-------->A

Where A catalyzes its own formation from X, then there is a saddle point at A = 0, except that of course negative values of A are impossible. If we make a reverse reaction going at a faster rate than the autocatalytic reaction for A, we can convert the saddle point into a fixed point, provided the reverse reaction saturates soon. Consider the molecule A in the original system. We add a reaction

EA

A------->A1

to remove A. The critical thing is to select rates for EA so that this gives a fixed point.

Let A'/A | A~0 = Kformation

That is, rate of production of A divided by amount of A when A is nearly zero = Kformation.

In our enzyme EA, rate of removal R = A.EA.k3/(A + Km)

Let R = 2 * A' when A ~ 0

Let EA saturate much below A_sts (A steady state)

Let the maximal rate for EA (at saturation) be much below other rates involving A.

Given these constraints, let

[EA] = Km = A_sts / 100

k3 = 2 * Kformation

Here the parameters 100 and 2 are adjustable. They are the saturation and speed respectively. We do not want them to be too big because that will make the system of equations very stiff and will adversely affect all numerical calculations.

Now we can remove A at the correct rate, but we need to get rid of A1. Turns out (by trial and error) that one way to do this is to redistribute A1 to each of the other molecules in the system in proportion to their concentrations. We obviously want to do this with minimal trapping of A1 in enzyme complexes. So, for a system with A, B and C we have:

AE A

A--------->A1-------->A

|

| B

---------->B

|

| C

---------->C

As AE is the rate-limiting step, we want the A, B, C steps to be fast. So, for each of them, kcat = 100, Km = 10, ratio = 4. We could avoid issues of enzyme-substrate complexes by using a classical Michaelis-Menten formulation, but with these rates the explicit complex form should not sequester much.

A final touch: With the above reaction scheme, we have inadvertently generated another saddle point, where all molecules are in state A1. To get rid of this, we can slowly remove A1 into A. Given the high speed of the other removal steps, a kb of ~0.02 should not affect our calculations much:

AE A

A--------->A1-------->A

<-------- |

kb | B

---------->B

|

| C

---------->C

Note that this complex set of reactions has been selected by trial and error to deal with a large number of reaction configurations. Often a smaller subset or alternative reactions will suffice. In our original example, the addition of the AE step alone can give rise to bistability, if we make A1 the same as X.

### Bistability analysis of a simple reaction

Consider the reaction system

b

a-------->b [Reaction 1]

a

b-------->a [Reaction 2]

Using Michaelis-Menten/Briggs-Haldane kinetics:

da/dt = a.b.kcat( ( 1 / (b + Km) ) - ( 1 / ( a + Km ) ) )

This is negative as a -> 0. Therefore any small amount of a will tend to decrease, going back to a ~0. By symmetry, the same will occur for b -> 0. So both points are stable, and we have bistability.

Using explicit representation of enzyme-substrate complex (mass-action kinetics) for the same two reactions:

k1A k3A

a + b <====> ea ----> 2a [Reaction 2]

k2A

k1B k3B

a + b <====> eb ----> 2b [Reaction 1]

k2B

Note that:

k1 = (k2 + k3)/Km

k3 = kcat.

Using quasi-steady-state assumption (OK as we approach a stable state):

ea ~ a.b.k1A / ( k2A + k3A ) = a.b/KmA

eb ~ a.b.k1B / ( k2B + k3B ) = a.b/KmB

then da/dt = [Reaction 2 terms for formation of a] - [Reaction 1 terms for removal of a]

= [ 2.k3A.ea - k1A.a.b + k2A.ea ] - [ k1B.a.b. - k2B.eb ]

Substituting for ea and eb.

= ea.(2k3A + k2A) – k1A.a.b + eb.k2B – k1B.a.b

= a.b.( 2k3A+k2A)/KmA – k1A.a.b + a.b.(k2B/KmB – k1B)

= a.b. [ { 2k3A + k2A – (k2A + k3A) }/KmA + (k2B – (k2B + k3B) )/KmB ]

= a.b. [ k3A / KmA – k3B/KmB ] [Equation 1]

Suppose Eq1 is negative. Then for any value of a > 0 and b > 0, da/dt is negative, and so the only stable root is at a = 0.

Conversely, the only stable root is b = 0 if Eq1 is positive.

In the interesting case where k3A/KmA = k3B/KmB, we have da/dt = db/dt = 0 for all values of a and b. This means that this is a line solution, that is, any value of a and b that satisfies the conservation laws will be a steady solution.

### Frequent motif mining

Viewing configurations as sets of reactions, we search for frequent motifs in a collection of systems by identifying frequently occurring subsets of reactions. We adopt the *Apriori* principle [5] wherein we enumerate (and evaluate) all possible candidate motifs bottom-up: beginning with individual reactions, followed by sets of 2-reactions, then sets of 3-reactions, and so on. The Apriori approach uses the downward closure property (also called *anti-monotonicity)* so that if a set of reactions is not frequent in the collection, then we can immediately prune out longer signatures that contain this set, since they will also be infrequent. During this process, we again take care to account for symmetry and isomorphism across different signatures.

We mine frequent motifs in each collection of bistable systems (3-mol, 4-mol, and 5-mol), pooling together all the instances of bistables found from the first as well as second phase of analysis. For 3-mol systems, we set a minimum frequency threshold of 0.01; for 4-mol systems, we use a threshold of 0.02; and for 5-mol systems, we use 0.05. Observe that by the definition of frequency, if a motif is frequent, all its subsets will also be identified as frequent motifs.

We search for the frequent motifs across all uncharacterized systems and rank the systems based on the number of motifs they exhibit. The top candidates are then considered for detailed characterization.

## References for Supplementary Information.

1. Sauro H, Ingalls B (2004). Conservation Analysis in Biochemical Networks: Computational Issues for Software Writers, *Biophys Chem.* 109: 1-15.

2. Nemeth A, Vidoczy T, Heberger K, Kuti Z, Wagner J (2002) MECHGEN: Computer Aided Generation and Reduction of Reaction Mechanisms. *J. Chem. Inf. Comput. Sci*. 42: 208-214.

3. Fic G, Nowak,G (2001) Implementation of similarity model in the CSB system for

chemical reaction predictions. *Computers and Chemistry* 25:177–186.

4. Sommese AJ, Wampler II CW (2005) *The Numerical Solution of Systems of Polynomials Arising in Engineering and Science*, World Scientific, Singapore.

5. Agrawal R, Srikant R (1994) Fast Algorithms for Mining Association Rules in Large Databases. In Mocca JB, Jarke M, Zaniolo C (Eds.) *Proceedings of the 20th International Conference on Very Large Data Bases (VLDB’94)*, 487-499.
